# Interocular symmetry of the peripapillary choroidal thickness and retinal nerve fibre layer thickness in healthy adults with isometropia

**DOI:** 10.1186/s12886-016-0361-7

**Published:** 2016-10-19

**Authors:** Mo Yang, Wei Wang, Quangang Xu, Shaoying Tan, Shihui Wei

**Affiliations:** 1 Department of Ophthalmology, Chinese PLA General Hospital, Fuxing Road NO.28, Beijing, Haidian District China; 2 Department of Ophthalmology, Shanxi Grand Hospital, Taiyuan, China; 3 Zhongshan Ophthalmic Center, State Key Laboratory of Ophthalmology, Sun Yat-Sen University, Guangzhou, China; 4 Department of Neurology, Chinese PLA General Hospital, Beijing, China

**Keywords:** Symmetry, Emmetropia, RNFL, Choroidalthickness, EDI SD-OCT

## Abstract

**Background:**

The aim of this study was to determine the interocular differences in the peripapillary retinal nerve fibre layer (RNFL), peripapillary choroidal thickness (PCT) and subfoveal choroidal thickness (SFCT) in healthy adults with isometropia, using enhanced depth imaging optical coherence tomography (EDI SD-OCT).

**Methods:**

One hundred healthy Chinese adults with spherical equivalents of ≤ ±3 dioptres and interocular differences of <0.5 dioptres were prospectively enrolled in this study. They underwent RNFL and PCT measurements via EDI SD-OCT, with a 3.4 mm scan circle centred on the optic nerve head. Subfoveal choroidal thickness (SFCT) measurements were also taken with a horizontal line scan centred on the macula. Right and left eyes were compared by a paired *t*-test, and the interocular differences were calculated. The agreement and correlations of the RNFLs, PCTs and SFCTs between the right and left eyes were analysed.

**Results:**

Eighty-six subjects (172 eyes) were included in the final analysis, consisting of 44 (51.6 %) males and 42 (48.8 %) females; 55 (63.9 %) had emmetropia and 33 (36.1 %) had ametropia. The RNFL was statistically significantly thicker in the right eyes when compared to the left eyes in the temporal quadrant, and thinner on average in the nasal superior quadrant (*p* < 0.05). However, the differences in the choroidal thicknesses in all of the quadrants between the right and left eyes were not statistically significant. The tolerance limits of the average RNFL were −21.1 μm and 7.1 μm, and the mean and standard deviation of the interocular difference in the average PCT was −2.2 ± 24.2 μm. The RNFLs and PCTs in all of the locations in the right eyes were significantly correlated with those in the left eyes. However, no significant associations between the age, sex, interocular asymmetry of spherical the equivalent or interocular differences in the RNFL and PCT were detected.

**Conclusion:**

The PCT did not differ significantly between the right and left eyes, although interocular asymmetry of the RNFL existed in this Chinese population with isometropia.

## Background

Because paired organs are not always perfectly symmetrical, an analysis of interocular symmetry can be a useful tool in clinical practice. Numerous studies have evaluated the role of asymmetrical parameters in various conditions, including the diagnosis of disease, detection of pathological abnormalities and prediction of disease progression. For example, interocular differences of greater than 2.0 in the cup to disc ratio have been shown to be a sign of glaucomatous damage, and have been widely used in clinical practice [1]. Similarly, other studies have reported that interocular asymmetry in the intraocular pressure (IOP) and retinal nerve fibre layer (RNFL) is associated with glaucomatous visual field defects [2, 3]. Moreover, in refractive or cataract surgery, high interocular symmetry of the spherical equivalent and visual acuity may be helpful in predicting the outcome of surgery on the fellow eye. In age-related macular degeneration, the severity in one eye affects the severity in its fellow eye [4].

Since the landmark study by Spaide and associates reporting choroid imaging in vivo with enhanced depth imaging using spectral domain optical coherence tomography (EDI SD-OCT), recent studies have focused on the measurement of choroidal thickness in healthy and disease conditions. In addition, it has greatly expanded our understanding of the pathogenesis of various retinal and optic diseases, such as age-related macular degeneration, pathological myopia, ischemic optic neuropathy and glaucoma [5–7].

Understanding the normal range of differences between eyes will help inform analysis of what degree of asymmetry between eyes can be considered possibly pathologic. Recently, several studies have detected the degree of interocular symmetry in the RNFL thickness and macular choroidal thickness in both adults and children, but the results were controversial [8–13]. The discrepancy may caused by different OCT devices, variation of inclusion criteria, ethnic variation, or different refractory status. These studies may also have been biased by the inclusion of subjects with isometropia and anisometropia. It was suggested that difference in refractive error between eyes would be expected to change the RNFL thickness and macular choroidal thickness [14, 15]. Moreover, no study has reported interocular differences in the peripapillary choroidal thickness (PCT) of healthy subjects. Therefore, the aim of the present study was to determine the range of interocular variation in the RNFL and PCT, as well as the factors associated with the interocular differences in normal Chinese adults with isometropia. This information may help to provide an indication of possible pathology, if there is some degree of asymmetry between a patient’s eyes.

## Methods

### Ethics

This prospective, cross-sectional study was conducted from July through August of 2015 in the Department of Neuro-ophthalmology at the General Hospital of the People’s Liberation Army (PLAGH) in China. This study adhered to the tenets of the Helsinki Declaration, and was approved by the PLAGH institutional review board. Written informed consent was obtained from all of the subjects before they entered the study.

### Subjects

Healthy adults were recruited from the staff, students and relatives of the patients at the PLAGH. The inclusion criteria in this study were as follows: 1) best corrected visual acuity (BCVA) on the Snellen chart of ≥1.0; 2) spherical equivalent (SE) of ≤ ±3.0 dioptres, astigmatism of <1.0 dioptre and interocular difference in the SE of < 0.5 dioptres (isometropia); 3) anterior segment and fundus appeared normal; 4) the RNFL thickness was within normal ranges. The SE was calculated by the sphere plus one-half of the cylinder degree. The adults with isometropia were further divided into emmetropia (SE ≤ ±0.5 dioptres) and ametropia (SE > ±0.5 dioptres). The major exclusion criteria were as follows: 1) intraocular pressure (IOP) ≥ 21 mmHg (Goldmann applanation tonometry); 2) interocular difference in the BCVA (Snellen chart) of > 0.1; 4) history of laser therapy, intraocular surgery or rigid contact lens wear. Those subjects with a history of diabetes, smoking, systemic hypertension, prematurity or other systemic diseases were also excluded from this study.

All of the subjects underwent complete ophthalmic evaluations, which included visual acuity measurements, slit-lamp biomicroscopy, gonioscopy, IOP measurements (Goldmann applanation tonometry), fundus examinations and refractive error examinations using an autorefractometer (KR-8900 version 1.07; Topcon Corporation, Tokyo, Japan).

### Optical coherence tomography imaging and analysis

**Fig. 1:**
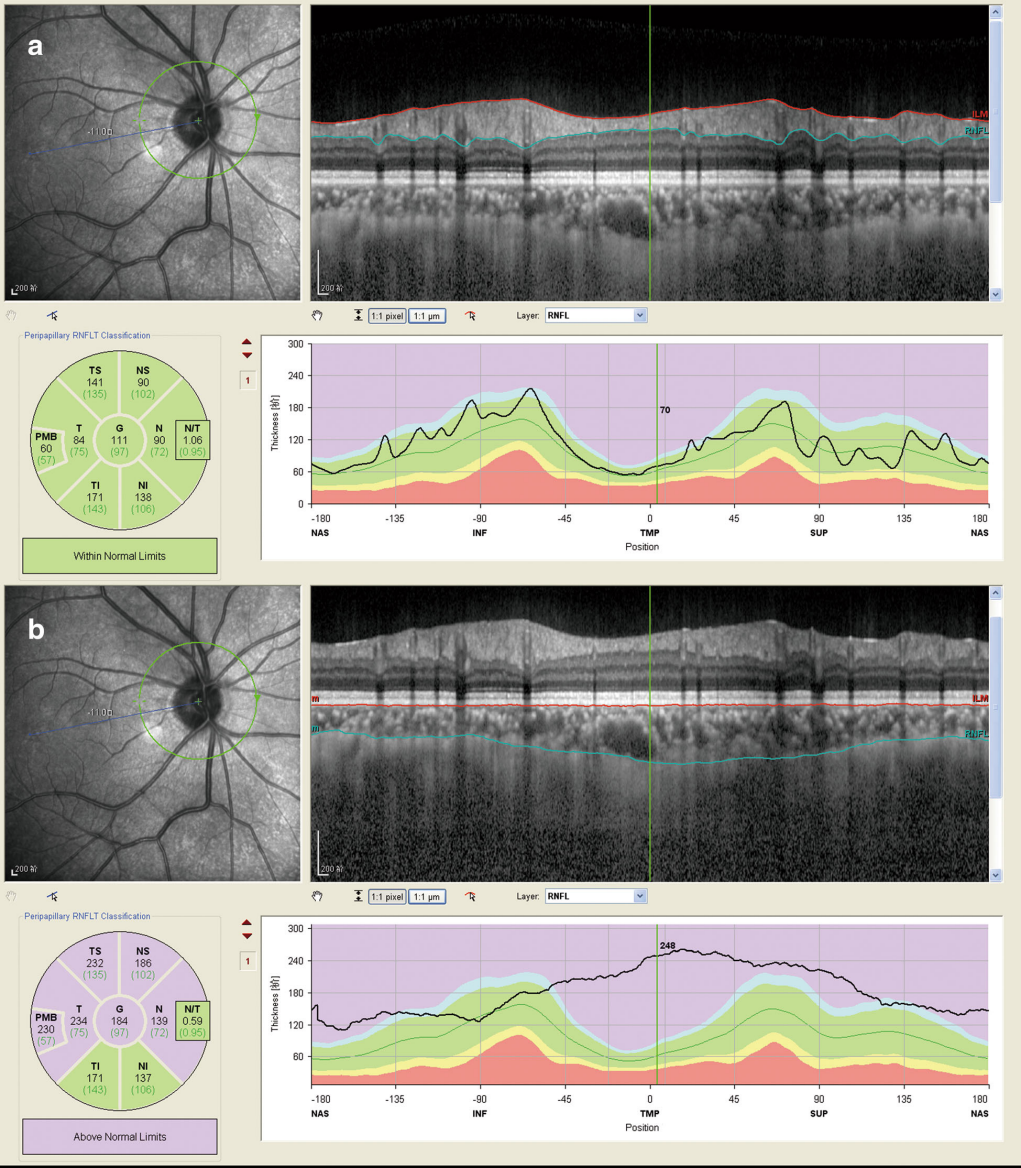
Illustration of retinal nerve fiber layer (RNFL) and peripapillarychoroidal thickness (PCT) measurements in isometropia eyes using enhanced depth imaging optical coherence tomography (EDI-OCT). **a** RNFL thickness in right eye; **b** PCT in right eye. The circle were segmented into six quadrants: nasal (N), temporal (T), nasal superior (NS), temporal superior (TS), nasal inferior (NI) and temporal inferior (TI)

All of the OCT scans were performed by the same experienced technician who was blind to the subject assignment. A commercial SPECTRALIS®SD-OCT (Heidelberg Engineering, Heidelberg, Germany) was used to obtain the RNFL and choroidal images, and the detailed methodology and standard protocol have been reported previously [16, 17]. Briefly, a 3.4 mm scan circle centred on the optic nerve head was used to obtain the RNFL and PCT measurements. In addition, a horizontal line scan centred on the macula was used to obtain the subfoveal choroidal thickness (SFCT) [17]. The RNFL values were automatically displayed for 6 quadrants (Fig. 1): nasal (N), temporal (T), nasal superior (NS), temporal superior (TS), nasal inferior (NI) and temporal inferior (TI). The RNFL and PCT measurements were obtained via SD-OCT in the enhanced depth imaging (EDI) mode.

The automatic averaging and eye-tracking features were used to better visualize the choroid, while the keratometry readings were entered into the Heidelberg machine to adjust for the magnification during the OCT examination. The resultant images were viewed and measured using Heidelberg Eye Explorer software (version 1.7.0.0; Heidelberg Engineering, Heidelberg, Germany). The choroidal thickness was measured manually as the distance between the inner edge of the retinal pigment epithelium and the outer aspect of the lamina fusca/inner border of the sclera. Like the RNFL, the PCTs in the six quadrants were presented after the identification of the two borderlines (Fig. 1). The averages of RNFL and PCT measurements were also calculated and used in the final statistical analyses. To avoid diurnal variations, all of the scans were obtained in the afternoon, between 17:00 and 20:00. Those subjects with inadequate image quality or segmentation failure in one or both eyes were excluded from the statistical analyses.

### Statistical analyses

All of the statistical analyses were performed using SPSS software (version 20.0; SPSS, Chicago, IL), and the data was presented as the mean and standard deviation (SD). Based on data from previous studies, we estimated that 26 healthy adults would be required to detect a significant difference in average PCT of at least 60.0 μm between right eyes and left eyes at a significance level of 0.05 and a power of 0.90, for a standard deviation of 40.3 μm [13, 15–17]. The normality of the parameter measurements was confirmed using a one sample Kolmogorov–Smirnov test, and the categorical variables were evaluated using Fisher’s exact test. The intraclass correlation coefficients (ICC) and Pearson’s coefficients were computed to measure the interocular agreement/correlation. Either the paired Student’s *t*-test or Wilcoxon paired test was used to compare the right eyes and left eyes, depending on whether normality could be assumed. Pearson’s correlation and a regression analysis were used to explore the relationship between the interocular differences (Δ, right eyes minus left eyes) between the RNFL or PCT and the other variables (e.g., age, sex, ΔSE). A *P*-value < 0.007 (0.05/7) was considered significant for associations for RNFL or choroidal thickness measurements. Otherwise, a *P* value of <0.05 was considered to be statistically significant.

## Results

A total of 100 healthy Chinese adults with isometropia were recruited for this research; however, 14 were excluded due to image artefacts or segment failure. Finally, 86 eligible subjects (172 eyes) were entered into the final statistical analyses. The mean age was 32.7 ± 11.7 years old (range 18 to 69), forty-four (51.6 %) were male, and 42 (48.8 %) were female. There were 55 (63.9 %) patients with emmetropia and 33 (36.1 %) with ametropia. The demographic characteristics and main clinical features of the right and left eyes are listed in Table 1. The mean SE was −0.65 ± 0.96 dioptres in the right eyes and −0.59 ± 0.93 dioptres in the left eyes (*P* = 0.123).

**Table 1:** Demographic and ocular characteristics of subjects

Characteristics	Mean ± SD or number (%)	*P*-value*
Number of subjects (eyes)	86 (172)	-
Age (range), year	32.7 ± 11.7 (18–69)	-
Gender (Female, %)	42 (48.8 %)	
Refractive status		
Emmetropia	55 (63.9 %)	
Ammetropia	33 (36.1 %)	
SE in all subjects, diopter		0.123
Right eye	−0.65 ± 0.96	
Left eye	−0.59 ± 0.93	

*SD* standard deviation, *SE* spherical equivalent

*P-value comparing right and left eyes by paired Student *t* test

Table 2 shows the data for the RNFLs and choroidal thicknesses in the right and left eyes, and their interocular differences. The RNFL in NS quadrant was thinner in the right eyes (120.3 ± 19.7 μm) than in the left eyes 133.9 ± 21.3 μm) (*P* < 0.001). Although the right eyes had thinner average RNFL (119.1 ± 11.1 versus 120.3 ± 11.2 μm, *P* = 0.034), it is worth noting that a difference in means of only one micrometer may not be particularly clinically significant. The right eyes had thicker RNFLs in the T quadrant and thinner RNFLs in the NS quadrant, when compared to the left eyes; however, no significant differences in the other quadrants were observed. With regard to the SFCT and PCT, highly interocular symmetry was observed. As Table 2 shows, the interocular differences in the SFCT and PCT in each quadrant were not significant (all *P* < 0.05). The 95 % confidence interval was −21.1 μm to 7.1 μm, respectively, depending on whether the RNFL was greater in the left eye or the right eye. The cut-off points for the average PCT were −74.1 μm and 46.4 μm.

**Table 2:** Measurements of peripapillary retinal nerve fibre layer thickness and choroidal thickness for right and left eyes and interocular difference

Parameters	Right eye		Left eye		Difference (right – left)				*P*-value*
	Mean	SD	Mean	SD	Mean	SD	Min	Max	
RNFL thickness									
TS, μm	156.6	19	154.1	20.5	2.5	15.2	−47	35	0.131
NS, μm	120.3	19.7	133.9	21.3	−13.6	15.7	−56	27	<0.001
N, μm	71.6	15	69.3	14.2	2.3	10.6	−40	27	0.051
NI, μm	122.9	22.7	122.2	23.4	0.7	17.9	−57	46	0.737
TI, μm	160.7	20.7	162.2	20.7	−1.4	12.3	−37	28	0.283
T, μm	82.3	11.9	80.1	13.2	2.2	8.7	−23	22	0.024
Average, μm	119.1	11.1	120.3	11.2	−1.2	5.4	−24.1	11.8	0.034
Choroidal thickness									
TS, μm	210.9	57.5	215.8	56.9	−4.9	32.8	−72	73	0.168
NS, μm	210.6	56.1	214.7	60	−4.1	33.5	−120	87	0.26
N, μm	198.6	58.3	202.3	62.6	−3.6	32.2	−118	94	0.299
NI, μm	175.1	61	176	58.8	−0.9	31	−108	70	0.792
TI, μm	176.4	61.1	175.4	58.9	1	33.3	−119	72	0.792
T, μm	208.3	65.1	208.6	62.7	−0.3	29.9	−85	73	0.917
Average, μm	196.7	55.7	198.8	55.8	−2.2	24.2	−93.9	49	0.411
SFCT, μm	353.6	80.2	359.1	81.6	−5.5	45.8	−109	124	0.274

*SD* standard deviation, *RNFL* retinal nerve fibre layer, *TS* temporal superior quadrant, *NS* nasal superior quadrant, *N* nasal quadrant, *NI* nasal inferior quadrant, *TI* temporal inferior quadrant, *T* temporal quadrant, *SFCT* subfovealchoroidal thickness

*P-value < 0.05 was considered significant

When the subjects were divided into emmetropia or ametropia subgroups, the analyses of interocular symmetry produced consistent results of RNFL thickness in the NS quadrant and choroidal thickness measurements (Table 3). Both emmetropes and ametropes showed asymmetry of RNFL thickness in NS quadrant. Significant interocular difference of RNFL thickness in the N quadrant was observed among emmetropia eyes, but not among ammetropia eyes. In addition, the interocular difference in the temporal quadrant exists only among emmetropia eyes and not ametropia eyes.

**Table 3:** Interocular differences of peripapillary retinal nerve fiber layer thickness and choroidal thickness in emmetropia and ametropia eyes

Parameters	Emmetropia (*n* = 55)				Ametropia (*n* = 31)			
	Mean ± SD	5th	95th	*P*-value	Mean ± SD	5th	95th	*P*-value
RNFL thickness								
ΔTS, μm	3.3 ± 12.5	−0.1	6.6	0.057	1.1 ± 19.2	−5.9	8.1	0.752
ΔNS, μm	−14.6 ± 14.9	−18.7	−10.6	<0.001	−11.7 ± 17.1	−18.0	−5.5	0.001
ΔN, μm	3.0 ± 9.6	0.4	5.6	0.023	0.9 ± 12.2	−3.6	5.4	0.683
ΔNI, μm	1.5 ± 17.2	−3.1	6.2	0.507	−0.9 ± 19.4	−8.1	6.2	0.79
ΔTI, μm	−1.7 ± 12.4	−5.0	1.7	0.328	−1.0 ± 12.2	−5.5	3.4	0.64
ΔT, μm	2.4 ± 8.1	0.2	4.5	0.034	1.8 ± 9.8	−1.8	5.4	0.32
ΔAverage, μm	−1.0 ± 4.9	−2.3	0.3	0.134	−1.7 ± 6.1	−3.9	0.6	0.142
Choroidal thickness								
ΔTS, μm	−3.6 ± 33.7	−12.7	5.5	0.427	−7.2 ± 31.6	−18.8	4.4	0.214
ΔNS, μm	−0.3 ± 33.7	−9.4	8.8	0.955	−10.9 ± 32.6	−22.9	1.1	0.073
ΔN, μm	−1.5 ± 27.7	−8.9	6	0.699	−7.5 ± 39.1	−21.8	6.9	0.295
ΔNI, μm	−1.2 ± 29.5	−9.2	6.8	0.764	−0.3 ± 34.0	−12.8	12.1	0.958
ΔTI, μm	0.9 ± 30.9	−7.4	9.3	0.825	1.0 ± 37.9	−12.9	14.9	0.884
ΔT, μm	−0.6 ± 29.5	−8.6	7.3	0.877	0.2 ± 31.1	−11.3	11.6	0.977
ΔAverage, μm	−1.0 ± 21.9	−7.0	4.9	0.727	−4.1 ± 28.0	−14.4	6.1	0.418
ΔSFCT, μm	−7.1 ± 42.9	−19.0	4.9	0.241	−3.0 ± 50.9	−21.6	15.7	0.748

*Δ* interocular difference (right eye minus left eye), *SD* standard deviation, *RNFL* retinal nerve fibre layer, *TS* temporal superior quadrant, *NS* nasal superior quadrant, *N* nasal quadrant, *NI* nasal inferior quadrant, *TI* temporal inferior quadrant, *T* temporal quadrant, *SFCT* subfovealchoroidal thickness

*P*-value < 0.05 was considered significant

The interocular correlations between the right and left eyes are shown in Table 4. Overall, the SFCT and PCT were highly correlated in both eyes, with ICCs of >0.9 for all of the measurements of choroidal thickness. The RNFLs of the homonymous quadrants were not correlated as well as the choroidal thicknesses were, although all of the ICCs for the RNFLs were >0.8.

**Table 4:** Agreement and correlations of retinal nerve fiber layer and choroidal thickness between right and left eye

Parameters	Pearson association*	ICC (95 % CI)*
RNFL thickness		
TS, μm	0.708	0.828 (0.736–0.888)
NS, μm	0.71	0.829 (0.738–0.888)
N, μm	0.737	0.848 (0.766–0.901)
NI, μm	0.698	0.822 (0.727–0.884)
TI, μm	0.825	0.904 (0.853–0.937)
T, μm	0.765	0.864 (0.792–0.912)
Average, μm	0.885	0.939 (0.907–0.96)
Choroidal thickness		
TS, μm	0.836	0.91 (0.862–0.942)
NS, μm	0.836	0.909 (0.861–0.941)
N, μm	0.86	0.924 (0.883–0.95)
NI, μm	0.867	0.928 (0.89–0.953)
TI, μm	0.846	0.916 (0.872–0.945)
T, μm	0.891	0.942 (0.911–0.962)
Average, μm	0.906	0.951 (0.924–0.968)
SFCT, μm	0.84	0.913 (0.865–0.944)

*ICC* intraclass correlation coefficient, *95%CI* 95 % confidential interval

*All *P* values < 0.001

Table 5 shows the results of the linear regression analysis of the interocular differences in the RNFLs and choroidal thicknesses. The interocular differences in the RNFLs were not correlated with the age or ΔSE. With regard to sex, no significant associations with the RNFL were detected, with the exception of the TS quadrant. In the interocular differences in the choroidal thickness, no correlations with the sex, ΔSE or age were noted (all *P* > 0.05), but the PCT and ΔNI were related to the age.

**Table 5:** Association between the interocular difference in retinal nerve fiber layer thickness and choroidal thickness with age, sex, and the interocular difference in spherical equivalent

Parameters	Age		Sex		ΔSE	
	β (95 % CI)	*P*-value*	β (95 % CI)	*P*-value*	β (95 % CI)	*P*-value*
RNFL thicknesss						
ΔTS, μm	−0.13 (−0.41 to 0.14)	0.339	8.26 (1.97 to 14.55)	0.011	−3.02 (−11.47 to 5.42)	0.479
ΔNS, μm	−0.01 (−0.30 to 0.28)	0.942	0.70 (−6.08 to 7.48)	0.837	1.93 (−6.85 to 10.71)	0.663
ΔN, μm	0.08 (−0.11 to 0.28)	0.393	0.06 (−4.52 to 4.64)	0.980	2.42 (−3.49 to 8.33)	0.418
ΔNI, μm	0.09 (−0.25 to 0.42)	0.610	−0.87 (−8.60 to 6.86)	0.824	4.36 (−5.62 to 14.33)	0.388
ΔTI, μm	−0.12 (−0.35 to 0.10)	0.288	4.28 (−0.94 to 9.49)	0.107	1.29 (−5.57 to 8.15)	0.709
ΔT, μm	−0.02 (−0.18 to 0.14)	0.781	1.18 (−2.55 to 4.91)	0.531	−2.84 (−7.64 to 1.97)	0.244
ΔAverage, μm	−0.02 (−0.12 to 0.08)	0.691	2.27 (0.01 to 4.53)	0.049	0.69 (−2.30 to 3.68)	0.648
Choroidal thickness						
ΔTS, μm	0.24 (−0.37 to 0.84)	0.433	−0.59 (−14.74 to 13.57)	0.935	8.32 (−9.93 to 26.57)	0.367
ΔNS, μm	0.04 (−0.58 to 0.66)	0.891	−0.74 (−15.19 to 13.71)	0.919	9.61 (−9.00 to 28.22)	0.307
ΔN, μm	−0.23 (−0.82 to 0.36)	0.445	5.19 (−8.66 to 19.05)	0.458	−5.62 (−23.58 to 12.35)	0.536
ΔNI, μm	−0.60 (−1.15 to −0.04)	0.037	8.19 (−5.07 to 21.44)	0.223	−2.14 (−19.46 to 15.19)	0.807
ΔTI, μm	−0.46 (−1.07 to 0.15)	0.141	5.49 (−8.85 to 19.83)	0.448	−2.38 (−21.02 to 16.26)	0.800
ΔT, μm	−0.15 (−0.70 to 0.40)	0.588	−2.99 (−15.87 to 9.90)	0.646	8.82 (−7.79 to 25.42)	0.294
ΔAverage, μm	−0.19 (−0.64 to 0.25)	0.394	2.45 (−7.96 to 12.86)	0.641	2.79 (−10.71 to 16.29)	0.682
ΔSFCT, μm	0.33 (−0.51 to 1.18)	0.435	−8.55 (−28.60 to 11.50)	0.399	11.65 (−13.96 to 37.26)	0.368

*Δ*, interocular difference (right eye minus left eye), *SE* spherical equivalent, *95%CI* 95 % confidential interval, *RNFL* retinal nerve fibre layer, *TS* temporal superior quadrant, *NS* nasal superior quadrant, *N* nasal quadrant, *NI* nasal inferior quadrant, *TI* temporal inferior quadrant, *T* temporal quadrant, *SFCT* subfoveal choroidal thickness

*P value <0.007 was considered significant

## Discussion

This study aimed to assess the interocular symmetry of the RNFL and PCT, as measured via EDI SD-OCT in an adult Chinese population with isometropia. We did not find a significant interocular difference in the PCT in any of the quadrants in the emmetropia or ametropia subgroups, and the normal limit for the interocular difference in the average PCT was 46.4 μm. With regard to the RNFL, we found that the interocular differences in the RNFLs for the average and two of the quadrants were statistically significant. Moreover, the interocular correlation was higher for the PCT than for the RNFL measurements. The interocular differences in the RNFLs and PCTs could not be explained by age, sex or the interocular differences in the SE.

Previous studies have focused on the physiological interocular differences in the RNFLs, but varied in their methodologies and populations [8–10, 18–31]. We found that the RNFL thickness in N and T quadrants was thicker in the right eyes for emmetropia subjects, and the RNFL thickness in the NS quadrant was thicker in the right eyes for both emmetropia and ametropia subjects. Our observations were in agreement with the recent studies [9, 10, 19, 20, 24, 27]. Using iVue100 OCT, Chen and colleagues showed that the RNFL was thinner in the right eyes in the NS for 2,324 young Chinese students [24]. Al-Haddad et al. [19] demonstrated that the RNFL thickness in the N and T quadrants showed thicker RNFLs in the right eyes, as measured via the high definition Cirrus OCT. Similarly, Park and associates reported that the RNFL thickness in N and T quadrants were thicker in the right eyes than that in left eyes for healthy Korean subjects using Stratus OCT [31]. We also found that the right eyes had similar average RNFL thickness compared to the left eyes, which is highly consistent with the previous studies [9, 10, 19, 20, 27]. Huynh found no significant interocular difference of average RNFL [30]. Chen and colleagues showed highly symmetrical in average in 2324 young Chinese students [24].

However, in a population-based study of 1,765 Australian children, Huynh found that the right eyes had significantly thicker RNFLs in the S and I quadrants, and thinner RNFLs in N and T quadrants [30]. Their observations were inconsistent with our results. This discrepancy may be associated with several factors: (1) the previous study included healthy children with mixed ethnicity, while we included only Chinese adults; (2) the old version Stratus OCT was used in their study, while the latest EDI SD-OCT was used to obtain RNFL measurements; (3) subjects with severe refractory errors were not excluded in their study, but strictly isometropia subjects were included in the present study.

There has been intense interest in the measurements of choroidal thickness in vivo in recent years, since the introduction of EDI SD-OCT [7, 32]. However, the interocular symmetry of the PCT has remained elusive. In this study, we found that the interocular differences in the PCT were not statistically significant, but large differences of up to 93.9 μm were observed at the individual level (Table 4). The choroid is a highly vascular structure with variable thickness regulated by various parameters including sympathetic nerve pathway and blood perfusion. An abnormal choroidal thickenss has been identified as indicator for several retinal and choroidal diseases [33]. The differences may related to interocular differences in blood perfusion. However, Rawji et al. [34] did not detect interocular difference in perfusion. Furthermore, the demographic factors and refractive status between both the eyes are highly similar in the present study. The difference at individual level may be a variation of normal phenomenon or underling indicators for some diseases, calling for further studies [15]. Though causative factors or significance of the large individual interocular differences was not identified currently, we believe further longitudinal studies may unveil the mystery.

One strength of this study was its strict inclusion of subjects with isometropia (ΔSE < 0.5 dioptres). Previous symmetrical analyses of the RNFL and macular choroidal thickness using EDI SD-OCT have included participants with large variations in the SE. Another strength of this study was its standardized protocol. To eliminate the factors that may affect choroidal circulation, those subjects with systemic or ocular disease, smokers and pregnant women were excluded, and the diurnal variation was controlled.

Nevertheless, the present study had some limitations. First, the majority of the subjects had emmetropia (SE ≤ ±0.5 dioptres), which may make the results less generalizable to patients with refractive error. Second, the lack of ethnic diversity limits the generalizability of our results. However, we believe that these findings can be extrapolated to other Asian populations. Third, all of the subjects were recruited from a tertiary care centre and underwent strict screening; therefore, the observed results may not be generalizable to the larger population. Forth, the PCTs were obtained semi-manually, which may introduce measurement bias, warranting future studies using a swept source OCT equipped with automatic segmentation software [35–37]. Finally, in addition to the age, sex and SE, other potential factors warrant investigation in future studies.

## Conclusions

In conclusion, this study explored the interocular differences in the RNFL and PCT in the adult Chinese population with isometropia. There was no significant difference in the PCT between the right and left eyes, but large degrees of asymmetry were observed at the individual level. In addition, the findings did show the interocular asymmetry of the RNFL. Future studies with larger populations and different ethnic groups are needed to confirm or refute the findings of the present study.

## Abbreviations

95 % CI: 95 % confidential interval
BCVA: Best corrected visual acuity
EDI-OCT: Enhanced depth imaging optical coherence tomography
ICC: Intraclass correlation coefficient
IOP: Intraocular pressure
N: Nasal quadrant
NI: Nasal inferior quadrant
NS: Nasal superior quadrant
PCT: Peripapillarychoroidal thickness
RNFL: Peripapillary retinal nerve fiber layer
RNFL: Retinal nerve fibre layer
SD: Standard deviation
SE: Spherical equivalent
SFCT: Subfovealchoroidal thickness
T: Temporal quadrant
TI: Temporal inferior quadrant
TS: Temporal superior quadrant
Δ: Interocular difference (right eye minus left eye)

## References

[1] Li H, Healey PR, Tariq YM, Teber E, Mitchell P (2013). Symmetry of optic nerve head parameters measured by the heidelberg retina tomograph 3 in healthy eyes: the Blue Mountains Eye study. Am J Ophthalmol.

[2] Hawker MJ, Vernon SA, Ainsworth G, Hillman JG, MacNab HK, Dua HS (2005). Asymmetry in optic disc morphometry as measured by heidelberg retina tomography in a normal elderly population: the Bridlington Eye Assessment Project. Invest Ophthalmol Vis Sci.

[3] Hollo G (2001). Intraocular and interocular symmetry in normal retinal capillary perfusion. J Glaucoma.

[4] Gangnon RE, Lee KE, Klein BE, Iyengar SK, Sivakumaran TA, Klein R (2015). Severity of age-related macular degeneration in 1 eye and the incidence and progression of age-related macular degeneration in the fellow eye: the Beaver Dam Eye Study. JAMA Ophthalmol.

[5] Wang W, Zhang X (2014). Choroidal thickness and primary open-angle glaucoma: a cross-sectional study and meta-analysis. Invest Ophthalmol Vis Sci.

[6] Zhang X, Wang W, Aung T, Jonas JB, Wang N (2015). Choroidal physiology and primary angle closure disease. Surv Ophthalmol.

[7] Spaide RF, Koizumi H, Pozzoni MC (2008). Enhanced depth imaging spectral-domain optical coherence tomography. Am J Ophthalmol.

[8] Hong SW, Lee SB, Jee DH, Ahn MD (2015). Interocular retinal nerve fiber layer thickness difference in normal adults. PLoS One.

[9] Lee SY, Jeoung JW, Park KH, Kim DM (2015). Macular ganglion cell imaging study: interocular symmetry of ganglion cell-inner plexiform layer thickness in normal healthy eyes. Am J Ophthalmol.

[10] Dalgliesh JD, Tariq YM, Burlutsky G, Mitchell P. Symmetry of retinal parameters measured by spectral-domain OCT in normal young adults. J Glaucoma. 2015;24:20–4. 10.1097/IJG.0b013e318287ac2f 23459201

[11] Ruiz-Medrano J, Flores-Moreno I, Pena-Garcia P, Montero JA, Duker JS, Ruiz-Moreno JM (2015). Asymmetry in macular choroidal thickness profile between both eyes in a healthy population measured by swept-source optical coherence tomography. Retina.

[12] Al-Haddad C, El CL, Antonios R, El-Dairi M, Noureddin B (2014). Interocular symmetry in macular choroidal thickness in children. J Ophthalmol.

[13] Chen FK, Yeoh J, Rahman W, Patel PJ, Tufail A, Da CL (2012). Topographic variation and interocular symmetry of macular choroidal thickness using enhanced depth imaging optical coherence tomography. Invest Ophthalmol Vis Sci.

[14] Hwang YH (2015). Factors affecting interocular differences in retinal nerve fiber layer thickness. Invest Ophthalmol Vis Sci.

[15] Kang HM, Kim SJ, Koh HJ, Lee CS, Lee SC (2015). Discrepancy in subfoveal choroidal thickness in healthy adults with isometropia. Ophthalmology.

[16] Huang W, Wang W, Zhou M, Chen S, Gao X, Fan Q, Ding X, Zhang X (2013). Peripapillary choroidal thickness in healthy Chinese subjects. BMC Ophthalmol.

[17] Wang W, Zhou M, Huang W, Chen S, Ding X, Zhang X (2013). Does acute primary angle-closure cause an increased choroidal thickness?. Invest Ophthalmol Vis Sci.

[18] Jee D, Hong SW, Jung YH, Ahn MD (2014). Interocular retinal nerve fiber layer thickness symmetry value in normal young adults. J Glaucoma.

[19] Al-Haddad C, Antonios R, Tamim H, Noureddin B (2014). Interocular symmetry in retinal and optic nerve parameters in children as measured by spectral domain optical coherence tomography. Br J Ophthalmol.

[20] Hwang YH, Song M, Kim YY, Yeom DJ, Lee JH (2014). Interocular symmetry of retinal nerve fibre layer thickness in healthy eyes: a spectral-domain optical coherence tomographic study. Clin Exp Optom.

[21] Field MG, Alasil T, Baniasadi N, Que C, Simavli H, Sobeih D, Sola-Del VD, Best MJ, Chen TC (2016). Facilitating glaucoma diagnosis with intereye retinal nerve fiber layer asymmetry using spectral-domain optical coherence tomography. J Glaucoma.

[22] Choi JA, Kim JS, Park HY, Park H, Park CK (2014). Retinal nerve fiber layer thickness profiles associated with ocular laterality and dominance. Neurosci Lett.

[23] Yamashita T, Sakamoto T, Kakiuchi N, Tanaka M, Kii Y, Nakao K (2014). Posterior pole asymmetry analyses of retinal thickness of upper and lower sectors and their association with peak retinal nerve fiber layer thickness in healthy young eyes. Invest Ophthalmol Vis Sci.

[24] Chen L, Huang J, Zou H, Xue W, Ma Y, He X, Lu L, Zhu J (2013). Retinal nerve fiber layer thickness in normal Chinese students aged 6 to 17 years. Invest Ophthalmol Vis Sci.

[25] Altemir I, Oros D, Elia N, Polo V, Larrosa JM, Pueyo V (2013). Retinal asymmetry in children measured with optical coherence tomography. Am J Ophthalmol.

[26] Sullivan-Mee M, Ruegg CC, Pensyl D, Halverson K, Qualls C (2013). Diagnostic precision of retinal nerve fiber layer and macular thickness asymmetry parameters for identifying early primary open-angle glaucoma. Am J Ophthalmol.

[27] Mwanza JC, Durbin MK, Budenz DL (2011). Interocular symmetry in peripapillary retinal nerve fiber layer thickness measured with the Cirrus HD-OCT in healthy eyes. Am J Ophthalmol.

[28] Qian J, Wang W, Zhang X, Wang F, Jiang Y, Wang W, Xu S, Wu Y, Su Y, Xu X (2011). Optical coherence tomography measurements of retinal nerve fiber layer thickness in chinese children and teenagers. J Glaucoma.

[29] Budenz DL (2008). Symmetry between the right and left eyes of the normal retinal nerve fiber layer measured with optical coherence tomography (an AOS thesis). Trans Am Ophthalmol Soc.

[30] Huynh SC, Wang XY, Burlutsky G, Mitchell P (2007). Symmetry of optical coherence tomography retinal measurements in young children. Am J Ophthalmol.

[31] Park JJ, Oh DR, Hong SP, Lee KW (2005). Asymmetry analysis of the retinal nerve fiber layer thickness in normal eyes using optical coherence tomography. Korean J Ophthalmol.

[32] Mrejen S, Spaide RF (2013). Optical coherence tomography: imaging of the choroid and beyond. Surv Ophthalmol.

[33] Ferrara D, Waheed NK, Duker JS (2016). Investigating the choriocapillaris and choroidal vasculature with new optical coherence tomography technologies. Prog Retin Eye Res.

[34] Rawji MH, Flanagan JG (2001). Intraocular and interocular symmetry in normal retinal capillary perfusion. J Glaucoma.

[35] Mansouri K, Medeiros FA, Marchase N, Tatham AJ, Auerbach D, Weinreb RN (2013). Assessment of choroidal thickness and volume during the water drinking test by swept-source optical coherence tomography. Ophthalmology.

[36] Park HY, Shin HY, Park CK (2014). Imaging the posterior segment of the eye using swept-source optical coherence tomography in myopic glaucoma eyes: comparison with enhanced-depth imaging. Am J Ophthalmol.

[37] Tan CS, Ngo WK, Cheong KX (2015). Comparison of choroidal thicknesses using swept source and spectral domain optical coherence tomography in diseased and normal eyes. Br J Ophthalmol.

